# New Appointments in the University of Edinburgh

**Published:** 1856-01

**Authors:** 


*New Appointments in the University of Edinburgh.*—The vacancy in the chair of natural history in this institution, arising from the death of Professor Forbes, has been filled by the appointment of Professor Allman, of Trinity College, Dublin. The professorship of the practice of medicine from which the veteran Professor Alison lately retired, has been given to Dr. Laycock, of London, whose appointment seems to meet with general approbation.— *Virginia Medical and Surgical Journal.*

